# Typological Diversity and Farm-System Vulnerability in Tropical Dual-Purpose Cattle Systems: A Case Study in Veracruz, México

**DOI:** 10.3390/ani16152393

**Published:** 2026-08-03

**Authors:** Elizabeth Zavala, Cecilio Barba Capote, Jorge Vieyra, Eva Boyer Bustamante, Antón García

**Affiliations:** 1 Doctoral Program in Natural Resources and Sustainable Management, University of Cordoba, 14071 Cordoba, Spain; pa2zamae@uco.es; 2 Iztapalapa Unit, Division of Biological and Health Sciences, Department of Reproductive Biology, UAM Autonomous Metropolitan University, Mexico City 14387, Mexico; jvieyra@yahoo.com; 3 Department of Animal Production, Faculty of Veterinary Sciences, University of Cordoba, 14071 Cordoba, Spain; pa1gamaa@uco.es; 4 International Doctoral School in Agri-Food (eidA3), University of Cordoba, Rabanales University Campus, 14071 Cordoba, Spain; v92bobue@uco.es

**Keywords:** dual-purpose systems, principal component analysis, livestock diversification, sustainability, bovine management practices

## Abstract

Dual-purpose cattle systems are among the most important livestock production systems in tropical regions, contributing significantly to food production and rural livelihoods. However, their considerable diversity in management practices, resource endowments, producer-reported climatic stressors, and production intensity remains insufficiently characterized for effective policy design. Therefore, this study aimed to identify and characterize farm typologies by integrating productive, structural, environmental, and management dimensions and to describe differences in farm-system vulnerability among them. Data were collected from 132 commercial farms using structured surveys. Twenty-three variables were analyzed using Principal Component Analysis of which 22 reached the predefined absolute loading threshold for component interpretation. The resulting component scores were subsequently used in the hierarchical cluster analysis. Seven principal components with eigenvalues > 1 were identified, explaining 68.87% of the total variance. Cluster 1 comprised extensive, milk-oriented farms with the lowest milk productivity but the greatest pasture availability and diversity; nevertheless, these farms were particularly affected by drought. Cluster 2 represented the predominant dual-purpose model, with intermediate productive, environmental, and diversification characteristics. Cluster 3 included more intensive and productive farms with limited land availability, the lowest soil conservation and pasture availability scores, high maintenance expenditure, and low investment capacity. Cluster 4 showed the highest milk productivity but also the greatest exposure to excessive rainfall and northerly winds. Accordingly, appropriate strategies should prioritize drought preparedness and forage conservation in Cluster 1, gradual improvements in efficiency and environmental management in Cluster 2, soil and pasture restoration and reduced maintenance dependence in Cluster 3, and climate-resilient infrastructure and risk-management measures in Cluster 4. These findings demonstrate that dual-purpose cattle systems are highly heterogeneous systems structured by multiple productive, environmental, and organizational dimensions. The results demonstrate that typology-specific interventions are more appropriate than uniform recommendations and provide a framework for improving productivity, sustainability, and climate resilience in tropical livestock systems.

## Simple Summary

Dual-purpose cattle systems are widely used in tropical regions because they produce both milk and meat while supporting the livelihoods of rural families. However, these farms show high heterogeneity in their resources, management practices, productivity, and ability to cope with environmental challenges. In this study, grouping farms with similar characteristics allows management recommendations to be better adapted to the needs of each production system. Four distinct farm types were identified, differing in production strategies, resource availability, environmental management, and dependence on external inputs. Understanding farm typologies can help researchers, advisors, and policymakers design more effective recommendations. Different farm types require different management strategies. This can improve productivity, sustainability, and resilience in tropical dual-purpose cattle systems.

## 1. Introduction

In Latin America, dual-purpose cattle systems (DPS) account for approximately 70% of the cattle herd in Mexico. The farmers manage crosses Bos taurus x Bos indicus herds fed predominantly by grazing with minimal concentrate supplementation [1,2]. These systems play a key role in regional food security and rural economies because of their flexibility, low external input requirements, and capacity to diversify production [3]. This advantage, even on small scales, allows producers to cope with the volatile economic conditions prevalent in the region [3,4]. On the other hand, DPS operates under highly variable environmental conditions, such as seasonal forage shortages, climate variability, limited access to water, and fluctuating market opportunities. As a result, producers have adopted diverse management practices and resource use strategies, generating a high degree of structural heterogeneity among production units due to differences in herd size, land availability, feeding practices, reproductive management, organization, technology adoption, and market integration [5]. This variability reflects differences in resource endowments and producers’ adaptive responses to local environmental and socioeconomic conditions. In Veracruz, specifically, these systems operate under a marked seasonal seasonality—six-month dry periods, irregular rainfall, and recurrent cold wind events (northerly winds)—necessitating adaptive management responses ranging from adjusting herd size to diversifying pasture.

From a system’s perspective, livestock production should be understood as a complex socio-ecological system in which productive management and adaptive capacity emerge from the interaction between social, technical, and environmental components [6,7,8]. In this context, the sustainability and resilience of livestock systems depend not only on biophysical resources but also on management strategies, diversification practices, family participation, and farmers’ capacity to respond to climatic and economic disturbances [9,10]. Challenges associated with climate change, resource degradation, and market pressures drive the growing need to move toward sustainable and resilient production models [10,11].

The heterogeneity of production systems has been extensively documented in studies conducted in tropical livestock systems, Previous research has shown that analysing these systems based on structure and productive indicators such as herd size, available land area, and production level is insufficient. While these variables provide useful information, they often oversimplify the complexity and heterogeneity of small livestock systems by neglecting key dimensions such as social organization, decision-making processes, labor structure, livelihood strategies, and the interaction between production and environmental conditions [12].

In this regard, DPS must simultaneously address improving productivity and sustaining rural livelihoods under conditions of increasing climate and resource scarcity. Therefore, understanding the differences between livestock farms in terms of resource management, production efficiency, and interaction with the environment is fundamental to transitioning to more resilient and sustainable production models.

Existing typologies of dual-purpose cattle systems have predominantly relied on productive and structural variables such as herd size, land area, and milk yield, while environmental management indicators (soil conservation, pasture diversity and tree cover) and input dependency remain underrepresented in multivariate classifications [13,14]. Consequently, farms with identical productivity profiles may differ substantially in ecological footprint and climate vulnerability yet receive identical policy prescriptions.

The marked structural, productive, and socioeconomic diversity of DPS makes it difficult to formulate homogeneous development strategies, thus requiring their study through typification approaches. Identifying typologies in DPS is relevant because it allows for the identification of different production strategies based on intensification levels, resource availability, and adaptability. This information enables the design of specific strategies, improves technology transfer processes, and supports decision-making at both the farm and institutional levels. Furthermore, typologies contribute to the evaluation of system sustainability by allowing for assessing social, productive, and environmental performance within comparable groups.

In this context, the climate vulnerability of tropical livestock systems depends on exposure to extreme weather events and the natural resource base that underpins the adaptive capacity of each farm. Studies conducted in silvopastoral and agroforestry systems in the Ecuadorian Amazon have documented that pasture diversity, tree cover, and soil conservation are the main components that differentiate resilient systems from those structurally fragile in the face of climate variability [15,16,17]. This evidence reinforces the need to incorporate environmental and diversification dimensions into the classification of livestock production systems beyond conventional production indicators.

Therefore, the objective of this study was to identify and characterize typologies of dual-purpose livestock systems by integrating productive, structural, environmental, and management variables and to analyse their farm-system vulnerability in relation to producer-reported climatic stressors, productive sensitivity, and adaptive capacity.

## 2. Materials and Methods

### 2.1. Population, Sample, and Survey

**Figure 1:**
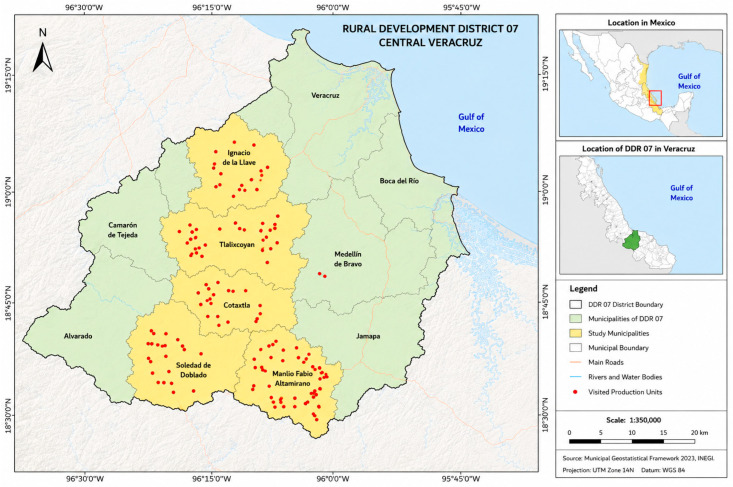
Geographic location of the farms studied in the State of Veracruz, Mexico.

The study was conducted in five municipalities: Cotaxtla (Cot), Ignacio de la Llave (I-Ll), Manlio Fabio Altamirano (MFA), Soledad de Doblado (SD), and Tlalixcoyan (Tla), belonging to Rural Development District 07 (RDD-07), located in the central region of the state of Veracruz, Mexico (Figure 1). The area is located at approximately 18°11′30″ N and 96°40′20″ W, with altitudes ranging from 0 to 320 m above sea level. According to its climatic characteristics, the area corresponds to a dry tropical climate (Aw), with mean annual temperature ranging between 16 °C and 40 °C. Rainfall is seasonal and concentrated during the summer months, generating marked dry periods, During the winter (December–March), strong northerly winds (“norths”) occur, with average speeds of 45 to 70 km h^−1^ and extreme gusts reaching up to 120 km h^−1^.

The study focused on commercial farms operating under mixed milk and beef production systems. A total of 132 farms were included in the study. Farms were selected from commercial dual-purpose cattle operations located in the five municipalities included in RDD-07. Eligibility criteria required farms to maintain both milk and beef production, to have been operating during the study period, and to provide complete information for the variables included in the analysis. Farms exclusively devoted to either dairy or beef production and farms with incomplete questionnaires were excluded. The farms were selected through non-probabilistic convenience sampling, based on farm accessibility, producer willingness to participate, and the availability of complete information. The sampling frame comprised accessible commercial dual-purpose cattle farms operating in the five municipalities included in RDD-07. As no complete and updated official census of the total dual-purpose cattle-farm population in RDD-07 was available, the sample cannot be considered statistically representative of the entire regional population. Accordingly, the findings should be interpreted as a characterization of the surveyed farms and generalized with caution. The questionnaire was administered face-to-face to the farm owner or the person primarily responsible for management decisions [3,5,13,18,19]. Seasonal production indicators, including dry-season milk yield (MKPD), were obtained from producers’ responses during the structured interview and reflected their recall of typical production conditions during the corresponding season. These indicators were not based on repeated direct measurements conducted by the research team. Standardized questions and clearly defined seasonal reference periods were used to improve response consistency. Nevertheless, some degree of recall bias and approximation error cannot be excluded. The questionnaire was developed from previous studies on the characterization of tropical dual-purpose cattle systems and was reviewed by researchers with experience in livestock production and farm typology analysis. Before the main survey, the questionnaire was pilot-tested on a small number of farms to assess question clarity, response consistency, and interview duration. Following the pilot phase, minor wording and ordering adjustments were introduced. The survey included variables grouped into three main categories: (i) production variables, including milk and beef production indicators; (ii) structural variables, including herd size, land use, labour availability, and stocking rate; and (iii) environmental and management variables, including forage availability, pasture and tree diversity, soil-conservation practices, feeding strategies, and animal diversification. The questionnaire used in this study is provided in the Supplementary Materials (Questionnaire S1). In total, 23 variables were considered for the PCA, of which 22 reached the predefined absolute loading threshold of 0.50 for component interpretation (Table 1). Several environmental, diversification, resource-availability, and impact variables were constructed from specific questionnaire items. Because these variables required additional explanation regarding their coding, aggregation, and interpretation, their construction is described in detail in Section 2.2 and Table 2.

### 2.2. Construction and Interpretation of Questionnaire-Derived Indices

The environmental diversification, resource-availability, and impact indices were derived from specific items of the structured questionnaire administered face-to-face to the farm owner or the person primarily responsible for farm-management decisions. The complete questionnaire and the items used for index construction are provided in Supplementary Material S1. Producers responded to categorical, multiple-response, and seasonal questions and did not directly assign scores from 1 to 10. The original responses were subsequently coded and combined to obtain the corresponding raw indicators.

The raw values obtained for each questionnaire-derived indicator were linearly rescaled to a common 1-to-10 range, where 1 corresponded to the minimum observed value and 10 to the maximum observed value. This transformation preserved the relative position of each farm within the original distribution and facilitated the joint inclusion of indicators expressed in different response formats in the multivariate analysis. The questionnaire items, construction criteria, scale anchors, and interpretation of each index are summarized in Table 2. The detailed coding procedures, item aggregation rules, raw score calculation, and transformation criteria used to derive each questionnaire-based indicator are provided in Supplementary Material S2.

All 132 questionnaires were administered face-to-face by the same trained interviewer using the same wording, response categories, and recording procedure. Therefore, inter-rater reliability was not applicable because the farms were not independently assessed by multiple interviewers. Procedural consistency was promoted through the use of a standardized questionnaire, expert review, pilot testing, and application of the same coding and transformation procedure to all observations.

### 2.3. Statistical Analysis

Before statistical analysis, the database was examined for missing values, data-entry errors, outliers, and inconsistencies. Farms with incomplete information for the variables included in the multivariate analysis were excluded. Because the variables were expressed in different measurement units, quantitative variables were standardized to zero mean and unit variance before principal component analysis [19]. Variable selection for the typological analysis combined statistical criteria, a structured literature review, and expert judgment, ensuring both analytical robustness and conceptual relevance to dual-purpose cattle systems [19,20]. Variables with a coefficient of variation below 60% or evidence of nonlinear dependence were excluded, following Niammuad et al. [20]. Variables were also assessed according to their conceptual relevance, potential redundancy, and contribution to the characterization of productive, structural, environmental, and management dimensions. Correlation and partial-correlation matrices were examined to identify redundant variables, and the dataset was subsequently reduced to 23 variables for the PCA. Of these, 22 reached the predefined absolute loading threshold of 0.50 on at least one retained component and were used for component interpretation. Principal Component Analysis (PCA) was then carried out to reduce dimensionality and identify the main sources of variability among farms. Component loadings were interpreted as indicators of the strength and direction of the relationship between each original variable and the retained components. Absolute component loadings equal to or greater than 0.50 were considered relevant for component interpretation. Each variable was assigned to the component on which it showed its highest loading, provided that the loading met this threshold. The number of retained components was determined using Kaiser’s criterion (eigenvalues > 1), and an orthogonal varimax rotation was applied to obtain a simpler and more interpretable loading structure while maintaining orthogonality among components. Sampling adequacy was evaluated using the Kaiser–Meyer–Olkin (KMO) index and Bartlett’s test of sphericity (*p* < 0.001). The overall KMO value was 0.701, indicating acceptable, although borderline, sampling adequacy for exploratory principal component analysis, and Bartlett’s test of sphericity was significant (*p* < 0.001) [18,21]. The communality of each variable was calculated as the sum of its squared loadings across the retained components and represents the proportion of its standardized variance accounted for by the retained PCA solution.

Standardized scores for the seven rotated principal components were calculated for each farm and used as input variables for the hierarchical cluster analysis. The DPS were then classified using cluster analysis. Hierarchical clustering was initially performed based on Ward’s method, using Euclidean, squared Euclidean, and Manhattan distances. The final clustering solution was selected by jointly considering the dendrogram, the Elbow criterion [22], the agglomeration-distance plot, within-cluster homogeneity, between-cluster separation, cluster size, and substantive interpretability. The four-cluster solution was retained because it provided the clearest and most meaningful differentiation among farm profiles. The Elbow criterion supported k = 4, as the reduction in within-cluster variation became substantially smaller beyond four clusters [23]. The agglomeration-distance plot was also examined to identify increases in fusion distance as progressively more heterogeneous groups were merged. The vertical axis of the dendrogram represents the linkage distance at which individual farms or groups of farms were merged. Because the cluster analysis was based on standardized component scores, this distance is dimensionless and does not correspond to a physical measurement unit. Internal cluster separation was additionally assessed using the average silhouette coefficient calculated from the seven retained principal component scores. Higher positive values indicate greater within-cluster cohesion and between-cluster separation. To further assess the robustness of the cluster solution, a linear discriminant analysis was performed using the seven retained principal component scores as predictor variables and cluster membership as the grouping variable. Classification performance was evaluated using both the original classification matrix and leave-one-out cross-validation.

In addition, the farms were characterized by their production orientation and level of socioeconomic development. Quantitative variables (Table 1) were analysed using one-way analysis of variance (ANOVA). Before ANOVA, the assumptions of normality and homogeneity of variances were assessed. When the overall ANOVA was significant, Tukey’s honestly significant difference test was used for pairwise comparisons among clusters. Eta-squared (η^2^) was calculated as an effect-size measure for each one-way ANOVA, representing the proportion of total variance in each quantitative variable associated with differences among clusters. Qualitative variables were compared using the Chi-square test. Categorical variables were compared among clusters using Pearson’s Chi-square test when the assumptions regarding expected frequencies were fulfilled. Expected frequencies were examined for all contingency tables, and when sparse cells were detected, with expected frequencies below five, Fisher–Freeman–Halton exact tests were applied. Degrees of freedom were reported for Pearson’s Chi-square tests. Cramer’s V was calculated as a measure of association strength, and adjusted standardized residuals were examined to identify the categories contributing to significant associations.

Farm-system vulnerability was interpreted through three analytical dimensions: producer-reported climatic stressors, productive sensitivity, and adaptive capacity. Producer-reported climatic stressors included the perceived effects of drought, rainfall, and northerly winds and were not interpreted as independent measures of climatic exposure. Productive sensitivity was interpreted from milk yield and questionnaire-derived indicators of pasture availability, pasture diversity, and soil and pasture management. Adaptive capacity was assessed from investment expenditure, management practices, diversification, resource availability, and dependence on external inputs. DILD was analysed separately as an animal-health burden indicator and was not considered a climatic stressor. Differences among clusters were evaluated using the corresponding quantitative variables rather than through a weighted composite vulnerability index. These dimensions were used to provide a descriptive farm-system vulnerability profile for each cluster.

All statistical analyses were performed using Statgraphics Centurion version XVI.1, Statgraphics Technologies, Inc., The Plains, VA, USA, (https://www.statgraphics.com/, accessed on 10 November 2025).

## 3. Results

### 3.1. Description of Typical Dual-Purpose Farms in Veracruz State

The main structural, socioeconomic, and management characteristics of the 132 dual-purpose cattle farms are summarized in Table 3. Overall, the farms were medium-sized, predominantly family-managed, and characterized by limited technological development and formalization.

These results indicate that dual-purpose cattle farming in the study area is primarily based on family labour, long-term farming experience, and local market participation. However, the low level of technological development, limited legal formalization, and restricted access to credit and government support may constrain investment capacity and technology adoption. Similar structural limitations have been reported for smallholder and dual-purpose cattle systems in Mexico [23,24,25,26,27,28].

### 3.2. Typology and Characterization of Dual-Purpose Cattle Systems

Table 4 summarizes the rotated component loadings by reporting the highest loading of each variable on the retained components. Seven components with eigenvalues greater than 1 were extracted, accounting for 68.87% of the explained variance [20]. Component loadings measured the relationship between the original variables and retained components and were used to interpret the meaning of the generated components. For interpretative purposes, each of the 22 variables that reached the predefined absolute loading threshold was assigned to the component on which it showed its highest absolute loading. The first three components accounted for 42.01% of the structural variability among dual-purpose cattle systems, which was concentrated in productive and intensification-related variables.

The first component was associated with meat production, accounting for 21.42% of the variance. The strongest loadings corresponded to the dimension related to meat productivity (MPC and MPH), sex ratio (SR), and annual concentrate intake per cow (CIC). This component distinguished extensive from semi-intensive meat production orientations.

The second component explained 10.71% of the variance and was associated with milk production. This component was strongly associated with annual milk production per cow (MKPC), dry-season milk production per cow (MKPD), and milk production per labour work unit (MKLWU). PC2 differentiated farms according to their milk-production performance within the dual-purpose systems. Livestock density (LUH) and milk productivity per unit area (MKPH) were the variables associated with the third factor, which explained 9.88% of the variance. A strong load was observed for livestock density, whereas the load was negative for milk yield per hectare. A strong positive loading was observed for livestock density, whereas milk production per hectare showed a negative loading.

The fourth component explained 8.42% of the variance through variables strongly associated with technical and economic attributes. High loadings were observed for land area per labour work unit, total pasture area, total herd size, and forage consumption per cow, indicating that farm size and resource endowment constituted a distinct structural dimension of differentiation.

The fifth component (7.23% of the variance) primarily reflected the biodiversity in the production systems through the diversification of livestock and non-livestock species. The remaining two components, together, explained 11.20% of the variance. These components were attributed to sustainability aspects (environmental management, MPC and EFP). Notably, PC7 (5.19%) indicated self-sufficiency of the production model, with a strong negative load for the percentage of external inputs and a positive load for pasture availability. This dimension differentiated self-sufficient, pasture-based systems from those dependent on purchased concentrates.

Finally, communalities, which estimated the proportion of variance in each variable explained by the retained components, are reported in Table 4.

**Figure 2:**
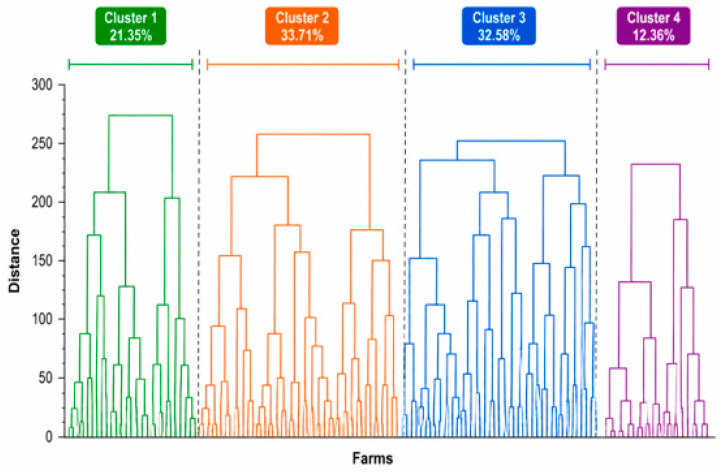
Dendrogram obtained from hierarchical cluster analysis showing the classification of the 132 dual-purpose cattle farms into four typologies. The coloured branches represent the farms assigned to each cluster, whereas the height at which branches merge indicates the linkage distance between individual farms or groups of farms. The vertical axis represents a dimensionless statistical measure of dissimilarity rather than a physical unit.

**Figure 3:**
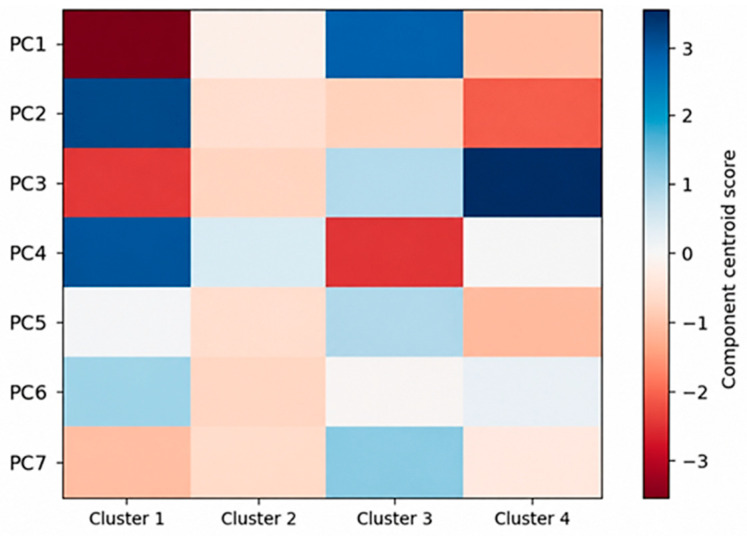
Heat map of the standardized centroid scores of the seven principal components across the four farm clusters. Positive scores indicate that a cluster is positioned above the overall sample mean for the corresponding component, whereas negative scores indicate a position below the overall mean. Cluster 1: Type I farms; Cluster 2: Type II farms; Cluster 3: Type III farms; and Cluster 4: Type IV farms.

The standardized scores of the seven retained principal components were used as input variables for the cluster analysis. Based on the 132 complete cases, four distinct clusters were obtained. The four-cluster solution is shown in the dendrogram in Figure 2, whereas the standardized cluster centroid scores are presented in the heat map in Figure 3. The agglomeration-distance plot provided in Supplementary S3 showed an increase in fusion distances as progressively more heterogeneous groups were merged. Considered together with the dendrogram, the silhouette coefficient, cluster sizes, and substantive interpretability of the resulting profiles, this pattern supported retention of the four-cluster solution. The robustness of the hierarchical clustering solution was further assessed through linear discriminant analysis based on the seven retained principal component scores. The original classification correctly assigned 92.13% of farms to their respective clusters, whereas leave-one-out cross-validation achieved an overall accuracy of 85.39%, supporting the consistency of the four-cluster solution [29]. The average silhouette coefficient for the four-cluster solution was 0.230, indicating positive but modest internal separation and some overlap among clusters. This result suggests that the identified typologies represent differentiated farm profiles within continuous productive, structural, and management gradients rather than completely discrete groups. The first comprised was 21.35%, the second was 33.71%, the third was 32.58%, and the fourth was 12.36% [23]. Figure 3 shows the centroid scores, which represent the standardized mean values of the selected variables for each cluster, summarizing the average profile of the farms within each group and allowing comparisons between clusters.

The dendrogram (Figure 2) shows how the farms group together hierarchically, while the heat map (Figure 3) displays the cluster centroid across the retained principal components, making it easier to see how the groups differ in terms of their principal components. In the dendrogram, farms or groups of farms that merge at lower linkage distances show greater similarity, whereas mergers occurring at higher linkage distances indicate greater dissimilarity. The coloured branches identify the four farm typologies retained in the final cluster solution. Taken together, the cluster analysis (Figure 3) and the characterization tables (Table 4) point to four distinct types of farms, each reflecting different, combinations of structural managerial and socioeconomic conditions in the sector.

Cluster 1 comprised 21.35% of the sample and included relatively large, extensive farms with a predominantly milk-oriented production strategy. Although these farms recorded the lowest milk yield per cow per year, they showed the greatest pasture availability and diversity, together with favourable soil conservation indicators. Cluster 2 comprised 33.71% of the sample and represented the predominant dual-purpose model, with intermediate values for milk yield per cow, resource availability, diversification, and environmental management. Cluster 3 comprised 32.58% of the farms and included more intensive operations characterized by relatively high milk yield per cow. However, these farms showed the lowest pasture availability and soil conservation and management scores, together with the highest maintenance expenditure and the lowest investment levels. Cluster 4 comprised 12.36% of the sample and recorded the highest milk yield per cow per year. These farms were also characterized by high investment levels and the greatest producer-reported impacts of rainfall and northerly winds.

Among the quantitative variables examined in the post-clustering characterization, eight showed statistically significant differences among the four clusters and are reported in Table 5. Although beef-production variables were included in the principal component analysis and contributed to the component structure used for clustering, they did not show statistically significant differences among clusters in the subsequent univariate characterization. Therefore, Table 5 reports only the quantitative variables that significantly differentiated the identified farm typologies. The largest effect sizes were observed for soil conservation and management (SMI; F = 17.77; *p* < 0.001; η^2^ = 0.385) and annual milk production per cow (MKPC; F = 17.28; *p* < 0.001; η^2^ = 0.379), indicating that these variables showed the strongest differentiation among the farm typologies. For MKPC, Cluster 4 recorded the highest mean value (1916 kg/cow/year), followed by Cluster 3 (1412 kg/cow/year), Cluster 2 (1047 kg/cow/year), and Cluster 1 (761 kg/cow/year). All four clusters differed significantly according to Tukey’s HSD test (*p* < 0.05). Regarding pasture resources, C1 showed the greatest diversity (2.47) and availability of pasture (9.39), both significantly higher than those of Cluster 3 (1.54 and 6.06, respectively; *p* < 0.001). Soil conservation and management followed the same pattern: Cluster 3 presented the lowest value (0.96), compared to C1 and C2, which did not differ (3.57 and 2.78; *p* < 0.001). As for the impact of climatic events, C4 was the most affected by excessive rainfall (1.97) and northerly winds (2.27), both differences being significant compared to C3 (0.17 and 1.04; *p* < 0.001 and *p* < 0.05, respectively). The disease rate was significantly lower in C3 (2.85%) compared to the other groups (4.52–4.78%; F = 9.20; *p* < 0.001). Finally, Cluster 3 showed the lowest investment expenditure (3.70), whereas Clusters 1 and 4 recorded significantly higher values (7.44 and 7.35, respectively; F = 4.82; *p* = 0.004). DILD (η^2^ = 0.245) and PAI (η^2^ = 0.237) also showed comparatively large differences among clusters, whereas DINW showed the smallest effect size among the quantitative characterization variables (η^2^ = 0.107).

Cluster 1 exhibited the greatest pasture diversity and availability, as well as the highest soil conservation and management score, but also reported a relatively high animal-health burden. Cluster 2 showed intermediate values for most variables. Cluster 3 recorded a higher milk yield per cow than Clusters 1 and 2, but a lower value than Cluster 4; it also showed the lowest pasture availability and soil conservation and management scores, the lowest producer-reported rainfall impact and animal-health burden, and the lowest investment expenditure. Cluster 4 recorded the highest milk yield per cow per year, the greatest producer-reported impact of northerly winds, a higher rainfall-impact score than Cluster 3, and high investment expenditure. Cluster 3 combined relatively high milk yield per cow with the weakest pasture and soil-management indicators and the lowest investment expenditure. Cluster 4 recorded the highest milk yield per cow and the greatest producer-reported impacts of rainfall and northerly winds. Cluster 1 recorded the lowest milk yield per cow but had the strongest pasture-resource base and the greatest reported impact of drought.

The Chi-square test revealed statistically significant differences between the four groups for three of the eight qualitative variables analyzed (Table 6). Age, sex, marital status, main source of income, and educational level did not significantly contribute to cluster differentiation. Significant differences were observed in the geographical distribution of farms. Cluster 1 and Cluster 4 were characterized by an overrepresentation of farms located in I-Ll, whereas Cluster 3 showed a marked underrepresentation of this municipality. Likewise, SD is underrepresented in Cluster 1 and Tlalixcoyan is underrepresented in Cluster 4. C1 was characterized by a significant overrepresentation of DPS (adjusted residual = 3.29), whereas Cluster 3 showed a significantly higher proportion of beef-oriented farms (adjusted residual = 2.33). Land tenure differed significantly among clusters. Cluster 3 was characterized by a lower presence of farms with owned land and a significantly higher proportion of farms with borrowed and rented land.

Based on the quantitative differences observed among clusters farm-system vulnerability was descriptively summarized through three analytical dimensions: producer-reported climatic stressors, productive sensitivity, and adaptive capacity (Table 7). Cluster 1 combined high drought exposure and high productive sensitivity with an intermediate adaptive capacity supported by a relatively favourable pasture base. Cluster 2 showed intermediate values across the three dimensions. Cluster 3 exhibited low direct exposure to climatic events but high productive sensitivity and low adaptive capacity because of poorer soil conservation, lower pasture availability, high maintenance expenditure, and limited investment. Cluster 4 combined high exposure to rainfall and northerly winds with low productive sensitivity and comparatively high adaptive capacity associated with its high productivity and investment level.

## 4. Discussion

The results demonstrate that the heterogeneity of dual-purpose cattle systems cannot be explained by a single productive or structural dimension. Instead, the principal-component structure revealed relatively independent gradients of productive orientation, intensification, structural scale, diversification, environmental management, and dependence on external inputs. This finding aligns with previous characterizations of DPS in Latin America, where livestock farms often shift their production strategies (meat or milk) depending on market access, input availability, and climatic limitations [2,4]. Furthermore, studies of tropical livestock system typologies have shown that production orientation and resource endowment are the main components determining variability among farms. According to the Food and Agriculture Organization of the United Nations, dual-purpose cattle systems represent a flexible strategy in tropical regions, allowing farmers to mitigate economic risk through product diversification [1].

PC3 identifies stocking rate as a major differentiating dimension. Stocking density has been widely recognized as a key driver of productivity, profitability, and environmental impact on grazing systems. The inverse relationship between livestock density and milk production per hectare suggests that increasing animal density may not result in proportional gains in dairy output, possibly because of resource competition, limited forage availability, or pasture degradation at higher stocking rates. These results are consistent with the literature indicating that intensification in production simultaneously increases vulnerability to climate stress and dependence on inputs [30,31]. In tropical DPS, moderate intensification has been associated with improved milk production per hectare. However, excessive stocking may reduce pasture recovery and system resilience [32].

PC4 aligns with typology studies of mixed livestock systems, which indicate that land availability and herd size are determining components in technology adoption and productivity levels. In Latin America, small- and medium-scale dual-purpose cattle systems typically operate under land constraints, which restrict mechanization, whereas larger farms may achieve economies of scale but can be more dependent on external inputs [33].

The diversification component (PC5) has been described as a key contributor to system resilience, since species diversification in livestock systems improves income variability and enhances nutrient cycling [34,35]. Studies conducted in silvopastoral systems in Colombia and Central America show that species diversity improves economic stability and food security for households [32]. It has also been identified as an adaptative response to climate variability in tropical regions [8].

The significant differences in production orientation, municipality, and land tenure, together with the absence of differences in most sociodemographic variables, suggest that the typology is driven primarily by productive and territorial conditions rather than by the personal characteristics of farm holders.

The vulnerability profiles should be interpreted as a comparative synthesis of the indicators analyzed rather than as the result of a single composite vulnerability index. The results of the present study reveal marked differences in farm-system vulnerability across the four identified farm clusters, a finding that aligns with the growing body of evidence emphasizing that the effects of climate change on livestock production systems are not uniform but vary according to agroecological context, production orientation, and resource endowment. Godde et al. [7] demonstrated that climatic variability and increasingly frequent extreme weather events are already slowing global agricultural productivity growth and threatening the provision of safe, nutritious, and affordable livestock products. Our data support this interpretation at the farm-system level: Cluster 4 combined the highest productivity with the greatest exposure to excessive rainfall and northerly winds, suggesting that high productive performance does not necessarily confer protection against climatic hazards and may reflect a trade-off between intensification and resilience. Thornton et al. [8] argued that the extent to which livestock systems are affected by climate change depends significantly on their adaptive capacity, and the higher investment levels recorded in C4 may represent a greater capacity to respond to these reported climatic stressors, though its long-term sufficiency remains uncertain.

Conversely, Cluster 3 presents a more complex farm-system vulnerability profile. Despite its relatively low reported exposure to extreme climatic stressors, Cluster 3 showed the weakest natural-resource indicators, the highest maintenance expenditure, and the lowest investment level This pattern is consistent with what Godde et al. [7] described as the indirect impacts of climate change on livestock supply chains, whereby cumulative deterioration or inadequate management of land and forage resources rather than discrete extreme events progressively undermines productive sustainability. Rojas-Downing et al. [36] documented that climate-driven stressors reduce milk production, animal growth, and fertility, generating significant economic losses for livestock producers. The elevated maintenance costs observed in Cluster 3 are consistent with this mechanism operating over time through resource depletion rather than acute shocks. In this sense, C3 can be characterized as exhibiting high productive sensitivity and low adaptive capacity, despite reporting relatively low impacts from the climatic stressors considered.

Cluster 1 combined the strongest pasture-resource base with the lowest productivity and the greatest reported impact of drought. This apparent paradox may reflect the well-documented sensitivity of extensive, forage-dependent systems to precipitation deficits. Noted that extreme weather events, such as droughts, rising temperatures, and unpredictable rainfall, are likely to adversely affect livestock production both in the short and long term, and C1’s low milk yield per cow in the context of adequate pasture resources suggests that producer-reported drought effects is already constraining the translation of resource availability into productive output [7]. Taken together, these findings underscore the importance of disaggregating farm-system vulnerability analyses at the farm-type level. Low adaptive capacity and high vulnerability to the climate emergency demand place-based adaptation responses, and the typology presented here provides a spatially and productively grounded basis for designing differentiated policies: investment support for Cluster 4 to buffer reported climatic stressors; soil and pasture restoration programs for Cluster 3; and drought management strategies for Cluster 1.

The patterns of differential vulnerability identified among clusters are consistent with the findings of Torres et al. [16,17] in silvopastoral systems along the Amazonian altitudinal gradient, where tree diversity and soil conservation showed the greatest explanatory power regarding the functional stability of the system in the face of environmental disturbances. Similarly, Torres et al. [16] demonstrated that existing natural capital pasture diversity, woody cover, and soil management practices constitute the main difference between producer groups with varying capacities to respond to climatic stresses, a result that directly corresponds to the discriminating role of soil conservation (F = 17.77; *p* < 0.001) and pasture availability variables identified in this typology. Taken together, this evidence from Latin American tropical livestock contexts underscore that climate resilience cannot be dissociated from the ecological basis of the production system and that typification approaches that integrate environmental dimensions, such as the one developed in this research work, build a robust framework for the design of differentiated interventions.

## 5. Conclusions

The study also identified contrasting farm-system vulnerability profiles among the four farm typologies, reflecting differences in producer-reported climatic stressors, productive sensitivity, and adaptive capacity. These profiles should be interpreted as a descriptive comparison among clusters rather than as externally validated levels of climate vulnerability.

The four typologies require differentiated management strategies. Cluster 1 would benefit from drought-preparedness measures, forage conservation, and improved water management to reduce the vulnerability of extensive, pasture-dependent farms. Cluster 2 requires gradual improvements in productive efficiency, diversification, and environmental management while maintaining its relatively balanced profile. Cluster 3 should prioritize soil restoration, pasture improvement, and the reduction in maintenance costs, as its productive performance is accompanied by a weak natural-resource base and limited investment capacity. Cluster 4 requires climate-resilient infrastructure, drainage, shelter against northerly winds, and risk-management measures to protect its high productivity from climatic exposure. From a policy perspective, extension services, financial support, and technology-transfer programmes should be designed according to farm typology rather than through uniform recommendations. Public interventions should combine productivity objectives with soil conservation, forage planning, self-sufficiency, and climate adaptation.

From a scientific perspective, future research should validate these typologies in other tropical regions and assess their stability over time. Longitudinal studies are also needed to examine whether farms move between typologies and to evaluate the effectiveness and economic feasibility of the proposed adaptation strategies. The integration of environmental, economic, and social indicators would further improve the assessment of sustainability and climate vulnerability in dual-purpose cattle systems. Accordingly, farms characterized by greater drought-related stress may benefit from water and forage-conservation strategies, whereas farms with low pasture availability and limited investment capacity may require support for soil and pasture restoration. More intensive farms reporting greater rainfall and northerly wind impacts may benefit from infrastructure and herd-management measures designed to reduce production disruption.

## Figures and Tables

**Table 1:** Dual-purpose cattle production system’s variables.

Typology Variables	Variable Description/Categories	Unit or Scale
MPC	Annual meat production per cow	kg/cow/year
MPH	Meat Production per hectare	kg/ha
SR	Herd sex ratio	females/males
CIC	Annual concentrate intake per cow	kg/cow/year
MKPC	Annual milk production per cow	kg/cow/year
MKPD	Milk production per cow during the dry season	kg/cow
MKLWU	Milk production per Labour Work Unit	kg/LWU
LUH	Livestock units per ha	Livestock units/ha
MKPH	Annual milk production per unit of farm area	kg/ha/year
LALWU	Land area managed per Labour Work Unit	ha/LWU
TPA	Total pasture area	ha
THS	Total herd size	Number of animals
FAC	Annual forage consumption per cow	kg/cow/year
PLS	Diversification of productive or working animal types other than cattle	1–10 index; higher values indicate greater livestock diversification
OWDI	Diversity of non-domesticated animal groups observed on the farm	1–10 index; higher values indicate greater observed wildlife diversity
MPF	Total annual meat production per farm	kg/farm/year
SMI	Extent of soil- and pasture-management practices implemented	1–10 index; higher values indicate broader implementation of soil and pasture management
TSD	Diversity of tree categories present on the farm	1–10 index; higher values indicate greater tree-type diversity
PSD	Diversity or richness of pasture types present on the farm	1–10 index; higher values indicate greater pasture richness
MPLWU	Meat production per Labour Work Unit	kg/LWU
EFP	Percentage of external feed inputs	Percentage of external feed inputs (%)
PAI	Continuity of pasture availability throughout the year	1–10 index; higher values indicate greater continuity of pasture availability
FCA	Forage crop area	ha
**Characterization** **indicators**		
DIR	Producer-reported adverse effects of rainfall or flooding	1–10 index; higher values indicate greater reported adverse impact
DINW	Producer-reported adverse effects of northerly winds	1–10 index; higher values indicate greater reported adverse impact
DILD	Reported disease burden and animal-health conditions	1–10 index; higher values indicate greater disease burden or poorer animal-health conditions
PEI	Proportion of expenditure allocated to farm investment	1–10 complementary expenditure score; higher values indicate a greater allocation to investment
Gender	Gender of the farm holder	Categorical: Man: (1); Woman: (2).
Municipality	Municipality in which the farm is located	Categorical: Cotaxtla (Cot), Ignacio de la Llave (I-Ll), Manlio Fabio Altamirano (MFA), Soledad de Doblado (SD), and Tlalixcoyan (Tla)
Education level	Highest educational level attained by the farm holder	Categorical: No formal education (1); Primary education (2); Secondary education (3); University education (4)
Marital status	Marital status of the farm holder	Categorical: Single (1); Common-law union (2); Married (3)
Source of income	Main source of household or farm-holder income	Categorical: Farm (1); Company employee (2); Merchant (3); Government employee (4); Self-employed professional (5)
Productive orientation	Main production orientation of the farm	Categorical: Increased meat production (1); Meat production equals milk production (2); Increased milk production (3)
Land tenure	Form through which the farm accesses the land	Categorical: Borrowed (1); Rented (2); Owned (3)
Land regime	Legal or institutional landholding regime	Categorical: Communal land (1); Ejido land (2); Small property (3)

**Table 2:** Questionnaire items, construction, and interpretation of the questionnaire-derived indices used in the multivariate analysis.

Index	Questionnaire Item(s)	Original Information	Construction and Interpretation
SMI	Q40	Soil- and pasture-management practices.	Higher values indicate broader soil and pasture management
PAI	Q39	Months with pasture available for animal feeding	Higher values indicate greater continuity of pasture supply throughout the year
PSD	Q38	Types of grasses present on the farm	Higher values indicate greater pasture richness
TSD	Q35–Q36	Presence of trees and reported three categories: fruit, forage, shade, and other trees	Higher values indicate greater tree-type diversity. This is not a formal species-diversity index
PLS	Q41	Other productive or working animal types present on the farm	Higher values indicate greater diversification beyond cattle
OWDI	Q42	Non-domesticated animal groups observed on the farm	Higher values indicate greater observed wildlife diversity
DIR	Q72 and Q74	Rainfall- or flood-related constraints, months considered as the rainy season, and reported effects on animals	Higher values indicate greater producer-reported adverse effects
DINW	Q72 and Q75	Northerly wind constraints, months considered as the northerly wind season, and reported effects on animals	Higher values indicate greater producer-reported adverse effects
DILD	Q82–Q83 and Q86	Internal and external deworming, diseases reported in the herd, treatment practices, and tick-control measures	Higher values indicate greater reported disease burden or poorer animal-health conditions

Questionnaire-derived indicators used for the characterization of dual-purpose cattle farms. Higher values indicate a greater presence or intensity of the evaluated characteristic, except for climatic-impact and animal-health burden indicators, for which higher values represent greater reported impacts. Detailed coding and aggregation procedures are provided in Supplementary Material S2.

**Table 3:** General characteristics of the dual-purpose cattle farms studied in Veracruz State.

Dimension	Indicator	Result
Farm structure	Average herd size	33.82 cows
Farm management	Family-management farms	58%
Labour	Average labour availability	1.5 FTE per farm
Farm infrastructure	Farms located near or integrated with the family home	90.5%
Housing characteristics	Homes built with traditional or mixed materials	62.9%
Basic services	Access to electricity	96%
Basic services	Water obtained from natural sources	81%
Basic services	Internet access	63%
Farm-holder profile	Male farm holders	84%
Farm-holder profile	Mean age of farm holders	52 years
Farm-holder profile	Married farm holders	86%
Education	Primary or secondary education	54.2%
Women’s participation	Mean age of participating women	41.4 years
Farm trajectory	Average time in operation	20 years
Income orientation	Farming as the main source of income	43%
Income orientation	Farming as a supplementary source of income	43%
Income orientation	Production mainly for self-consumption	14%
Formalization	Farms without formal legal registration	82%
Market orientation	Main products	Milk and beef
Market orientation	Main market destination	Local markets
Technology	General level of technological development	Low
Financial access	Access to credit and public support	Limited

**Table 4:** Summary of the highest rotated component loading and communalities for the variables included in the PCA.

Variable	Loading	Communality	Eigenvalue	Explained Variance (%)	Cumulative Variance (%)	PC
MPC	−0.76	0.70	4.93	21.42	21.42	1
MPH	−0.66	0.64				
SR	−0.64	0.58				
CIC	−0.59	0.50				
MKPC	0.85	0.78	2.46	10.71	32.13	2
MKPD	0.84	0.77				
MKLWU	0.68	0.76				
LUH	0.85	0.86	2.27	9.88	42.01	3
MKPH	−0.79	0.76				
LALWU	0.84	0.84	1.94	8.42	50.43	4
TPA	0.80	0.81				
THS	0.68	0.83				
FAC	0.50	0.71				
FCA	−0.38	0.49				
PLS	0.73	0.56	1.66	7.23	57.66	5
OWDI	−0.64	0.61				
MPF	0.53	0.62				
SMI	0.73	0.70	1.38	6.01	63.68	6
TSD	0.64	0.55				
PSD	0.61	0.65				
MPLWU	−0.54	0.55				
EFP	−0.82	0.74	1.19	5.19	68.87	7
PAI	0.57	0.64				

**Table 5:** Characterization of dual-purpose cattle farm typologies according to quantitative variables.

Variable	C1	C2	C3	C4	F	*p*	η^2^
MKPC ^1^	761.37 ^a^	1046.97 ^b^	1412.24 ^c^	1916.36 ^d^	17.28	<0.001	0.37
PSD	2.47 ^a^	1.75 ^b^	1.54 ^b^	2.24 ^ab^	5.11	0.003	0.15
PAI	9.39 ^a^	8.86 ^a^	6.06 ^b^	8.64 ^a^	8.82	<0.001	0.23
SMI	3.57 ^a^	2.78 ^a^	0.96 ^b^	2.52 ^a^	17.77	<0.001	0.38
DIR	1.14 ^a^	1.11 ^a^	0.17 ^b^	1.97 ^a^	6.86	<0.001	0.19
DINW	1.58 ^ab^	1.72 ^ab^	1.04 ^b^	2.27 ^a^	3.38	0.022	0.10
DILD	4.79 ^a^	4.52 ^a^	2.86 ^b^	4.63 ^a^	9.20	<0.001	0.24
PEI	7.44 ^a^	5.98 ^ab^	3.70 ^b^	7.35 ^a^	4.82	0.004	0.14

Different superscript letters (a, b, c, d) within the same row indicate significant differences according to Tukey’s HSD test (*p* < 0.05); means sharing at least one letter do not differ significantly. Exact ANOVA *p*-values are reported in the *p* column, and eta-squared (η^2^) indicates the proportion of variance associated with differences among clusters. ^1^ MKPC denotes annual milk production per cow and is referred to in the text as milk yield per cow.

**Table 6:** Characterization of dual-purpose farms according to qualitative variables.

Variable	Test	Statistic	df	*p*-Value	Cramer’s V
Gender	Pearson χ^2^	4.32	3	0.22	0.220
Municipality	Fisher–Freeman–Halton	26.20	-	0.01	0.313
Education level	Fisher–Freeman–Halton	58.29	-	0.05	0.467
Marital status	Fisher–Freeman–Halton	3.31	-	0.34	0.193
Source of income	Fisher–Freeman–Halton	5.15	-	0.55	0.170
Productive orientation	Pearson χ^2^	8.84	3	0.03	0.313
Land tenure	Fisher–Freeman–Halton	17.23	-	0.01	0.311
Land regime	Pearson χ^2^	4.38	3	0.22	0.222

Pearson’s Chi-square tests are reported with degrees of freedom (df). Fisher–Freeman–Halton exact tests were applied when expected frequencies below five were identified. Cramer’s V indicates the strength of association.

**Table 7:** Farm-system vulnerability profile by cluster.

Cluster	Producer-Reported Climatic Stressors	Productive Sensitivity	Adaptive Capacity
C1	High (drought)	High (lowest productivity)	High (strong pasture base, soil and pasture management, and high investment)
C2	Medium	Medium	Medium
C3	Low (low reported impacts across climatic stressors)	High (low pasture availability and weak soil and pasture management)	Low (high maintenance, low investment)
C4	High (rainfall, northerly winds)	Low (highest milk yield per cow and relatively high pasture availability)	High (high investment and relatively high pasture availability)

High, medium, and low categories summarize the relative position of each cluster based on cluster means and statistically significant differences reported in Table 7. These categories do not represent externally validated thresholds or a weighted composite vulnerability index.

## Data Availability

The data presented in this study are available from the corresponding author upon reasonable request.

## Supplementary Materials

The following supporting information can be downloaded at: https://www.mdpi.com/article/10.3390/ani16152393/s1, Supplementary Material S1: Questionnaire used for data collection; Supplementary Material S2: Definition, coding, and aggregation rules of questionnaire-derived indicators; Supplementary Material S3: Additional statistical analyses supporting the robustness and characterization of the farm typologies. Figure S1: Agglomeration-distance plot obtained from hierarchical cluster analysis using Euclidean/squared Euclidean distance.

## Abbreviations

The following abbreviations are used in this manuscript:

ANOVA	Analysis of Variance
CIC	Concentrate intake per cow per year
Cot	Cotaxtla
DILD	Degree of impact from livestock diseases
DINW	Degree of impact from northerly winds
DIR	Degree of impact from rainfall
DPS	Dual-purpose cattle systems
EFP	Percentage of external feed inputs
FAO	Food and Agriculture Organization of the United Nations
FAC	Forage availability per cow per year
FTE	Full-time equivalent
HSD	Honestly significant difference
I-Ll	Ignacio de la Llave
KMO	Kaiser–Meyer–Olkin
LALWU	Land area managed per labour work unit
LU	Livestock unit
LUH	Livestock units per hectare
LWU	Labour work unit
MFA	Manlio Fabio Altamirano
MKPC	Milk production per cow per year
MKPD	Milk production per cow during the dry season
MKPH	Milk production per hectare per year
MKLWU	Milk production per labour work unit
MPC	Meat production per cow per year
MPF	Meat production per farm per year
MPH	Meat production per hectare
MPLWU	Meat production per labour work unit
PAI	Pasture availability index
PC	Principal component
PCA	Principal component analysis
PEI	Proportion of expenditure dedicated to investment
PLS	Presence of other livestock species index
PNLS	Presence of other non-livestock productive species index
PSD	Pasture species diversity index
RDD	Rural Development District
SD	Soledad de Doblado
SIAP	Servicio de Información Agroalimentaria y Pesquera
SMI	Soil conservation and management index
SR	Herd sex ratio
THS	Total herd size
Tla	Tlalixcoyan
TPA	Total pasture area
TSD	Tree species diversity index
UAM	Universidad Autónoma Metropolitana
