# Supplementary material for: Typological Diversity and Farm-System Vulnerability in Tropical Dual-Purpose Cattle Systems: A Case Study in Veracruz, México
Source: Animals (Basel). 2026 Aug 3;16(15):2393. doi: 10.3390/ani16152393 (PMC13464325; doi:10.3390/ani16152393)
Supplement: Supplementary file 1 [file animals-16-02393-s001.zip › animals-4451602-supplementary.pdf]

Supplementary Material S1: Questionnaire used for data collection

Survey No. _______

Metropolitan Autonomous University. Mexico

DUAL-PURPOSE CATTLE QUESTIONNAIRE

This questionnaire is for informational purposes and is part of an academic research project aimed at identifying socioeconomic, technological, and environmental indicators developed by producers in the central region of the state of Veracruz. The information obtained will be used for academic research purposes and will not be disclosed individually.

I. IDENTIFICATION OF THE PRODUCER

1. Producer's Name ____Sex F ( ) M ( )

2. Address ____Locality __________________
   Municipality ____State __________________

3. Ranch geolocation ________________________ Phone number (optional) ________________________

4. Age ___________

5. How many years of schooling? ___________

6. How many years have you been a livestock producer? ___________

7. Marital status (Mark with an X)

1) Single	2) Married	3) Common-law union	4) Other

8. What is your main source of income? (Mark with an X)

1) The ranch	2) Company employee	3) Government employee	4) Merchant	5) Other

9. Do you carry out any other economic activity besides livestock farming? (Mark with an X)

0) None	1) Agricultural day laborer	2) Government employee	3) Merchant	4) Other

10. You produce (Mark with an X)

1) Meat and milk
2) Cow-calf (breeding)
3) Milk only
4) Feeder calves

11. Feeder calves
    How long does it take to finish them? ___________
    What is the finishing weight? ___________
    How are the animals sold? (Mark with an X)

1) By kilogram	2) By lot/batch	3) Other

12. Do you consider yourself a dual-purpose producer? Yes ( ) No ( )

Supplementary Material S1: Questionnaire used for data collection

II. TECHNICAL-PRODUCTIVE PROFILE

13. Type of land tenure? (Mark with an X)

1) Owned	2) Borrowed/loaned	3) Rented

14. Land tenure regime (Mark with an X)

1) Small private property	2) Ejido (communal agrarian land)	3) Communal

15. What is the total surface area of the ranch (ha)? ____________

16. Distribution of the surface area

Land use category	Area (ha)
Grazing pastures	
Rangeland pastures	
Cut forage production	
Forage crops	
Silos	
Facilities (corrals and warehouses)	
Other	

17. What services are available on the ranch? (You may select more than one)

0) None	1) Drinking water	2) Electricity
3) Sewage/drainage	4) Internet	5) Other

18. Corrals and type of material? (You may select more than one)

0) No corral(s)	1) Wood	2) Wire
3) Iron	4) Concrete	5) Other

19. Livestock inventory (previous year)

Type of animal	No. of animals	No. of animals that died last year
Total adult cows		
Cows in production		
Heifers (from service to first calving)		
Females in development (weaning to service)		
Female calves		
Male calves		
Young bulls		
Breeding bulls		

20. The breeding bull(s) is/are used: (Mark with an X)

0) No breeding bull(s)	1) Exclusive use	2) Shared
3) Rented/borrowed	4) Other	

Supplementary Material S1: Questionnaire used for data collection

21. Do you have purebred animals? Yes ( ) Which breed? ________________________________ No ( )

22. Which crossbreeds predominate in your herd? ____

23. How many months is the cow milked? ___________

24. How many days after calving does the cow return to service (rebreeding interval)? ____________

25. Number of milkings per day per cow ____________

26. How many days did you milk last year? ____________

27. How many liters of milk does the herd produce on average per season? Dry season _______ Rainy season _______

28. What is the selling price per liter of milk? Dry season _______ Rainy season _______

29. How long does milking take per day? ____________

30. Productive indicators

Indicator	Value
Weaning age of animals (months)	
Weaning weight of calves (kg)	
Birth weight of calves (kg)	

31. Reproductive indicators

Indicator	Value
Number of cows that became pregnant last year	
Number of calves born last year	

32. Reproduction method? (Mark with an X)

1) Free/natural mating	2) Artificial insemination	3) Controlled mating	4) Other

III. ENVIRONMENTAL

33. Do you know / are you aware of what climate change is? Yes ( ) No ( ) (if No, skip to question 34)

34. Do you take any measures to mitigate the environmental effects of your production? __________________________

1. Biodiversity of flora and fauna

35. Do you have shade trees on the ranch? Yes ( ) No ( )

36. Type of trees on the ranch (Mark with an X)

0) No trees	1) Fruit trees	2) Forage trees	3) Shade trees	4) Other

37. Feeding system (Mark with an X)

1) Grazing	2) Grazing and supplementation	3) Other

38. What grass(es) do you have, and where do you obtain the seed? (Mark with an X)
Seed source:

0) No grass	0) Does not buy seed	1) Feed store / Veterinary supplier	2) Distributor
3) Livestock association	4) Grass transplant	5) Other	

Supplementary Material S1: Questionnaire used for data collection

Type of grass:

1) Star grass	2) Pangola	3) Humidicola	4) Tanzania
5) Mulato	6) Llanero	7) Insurgente	8) Taiwan grass
9) King grass	10) Cobra	11) Señal	12) Mombaza
13) Other			

39. In which months do you have grass available to feed the animals? (Mark with an X)

Jan	Feb	Mar	Apr	May	Jun	Jul	Aug	Sep	Oct	Nov	Dec

40. Soil conservation practices (Mark with an X)

0) None	1) Manure incorporation	2) Silvopastoral system
3) Pasture rotation	4) Electric fencing	5) Grass species diversity
6) Pasture maintenance (fertilization, weeding, burning)	7) Stocking-rate control	8) Chemical fertilization
9) Other		

41. What other productive or working animals do you have on the ranch? (Mark with an X)

0) None	1) Poultry	2) Pigs	3) Bees
4) Horses	5) Mules	6) Donkeys	7) Other

42. Non-productive (wild) species observed on the plot? (Mark with an X)

0) None	1) Rodents (rabbits, mice, rats, etc.)	2) Lizards
3) Birds	4) Snakes	5) Bats
6) Beetles	7) Iguanas	8) Other

2. Use of external inputs

43. How often do you offer concentrate feed to the animals? (Mark with an X)

0) Does not offer (skip to question 47)	1) All year	2) Dry season
3) Rainy season	4) Only when money is available	5) Other

44. Amount of concentrate feed purchased (during one year) ____________

45. Type of animals given concentrate feed (Mark with an X)

1) All animals in the herd	2) Dry cows	3) Milking cows	4) Weaned calves
5) Heifers or young bulls	6) Breeding bulls	7) Feeder calves	8) Other

46. Where do you obtain the concentrate feed? (Mark with an X)

1) Locality	2) Another locality within the municipality	3) Outside the municipality
4) Another State	5) Other	

47. How often do you offer mineral salts to the animals? (Mark with an X)

0) Does not offer (skip to question 51)	1) All year	2) Dry season
3) Rainy season	4) Only when money is available	5) Other

48. Type of animals given mineral salts (Mark with an X)

1) All animals in the herd	2) Dry cows	3) Milking cows	4) Weaned calves
5) Heifers or young bulls	6) Breeding bulls	7) Feeder calves	8) Other

49. Amount of mineral salts purchased last year ____________

50. Where do you obtain the mineral salts? (Mark with an X)

1) Locality	2) Another locality within the municipality	3) Another municipality
4) Another State	5) Other	

51. How often do you offer silage to the animals? (Mark with an X)

0) Does not offer (skip to question 55)	1) All year	2) Dry season
3) Rainy season	4) Only when money is available	5) Other

52. Amount of silage offered (during one year) ____________

53. Type of animals given silage (Mark with an X)

1) All animals in the herd	2) Dry cows	3) Milking cows	4) Weaned calves
5) Heifers or young bulls	6) Breeding bulls	7) Feeder calves	8) Other

54. Where do you obtain the silage? (Mark with an X)

1) Own production	2) Locality	3) Another locality within the municipality
4) Another municipality	5) Another State	6) Other

55. How often do you offer hay or bales to the animals? (Mark with an X)

0) Does not offer (skip to question 59)	1) All year	2) Dry season
3) Rainy season	4) Only when money is available	5) Other

56. Amount of hay or bales offered (during one year) ____________

57. Type of animals given hay or bales (Mark with an X)

1) All animals in the herd	2) Dry cows	3) Milking cows	4) Weaned calves
5) Heifers or young bulls	6) Breeding bulls	7) Feeder calves	8) Other

58. Where do you obtain the hay or bales? (Mark with an X)

1) Own production	2) Locality	3) Another locality within the municipality
4) Another municipality	5) Another State	6) Other

59. What type of fertilization do you use? (Mark with an X)

0) Does not use (skip to question 61)	1) Manure	2) Chemical fertilizer
3) Biofertilization (algae, mycorrhizae, etc.)	4) Other	

60. Type of agrochemical containers, and disposal method (Mark with an X)

Container type:

1) Glass	2) Aluminum	3) Sheet metal

4) PET	5) Plastic	6) Other

Disposal method:

1) Trash	2) Open dump	3) Special deposit sites	4) Left in the field
5) Reused	6) Burned	7) Other	

61. How often do you use herbicides per year? (Mark with an X)

0) Does not use (skip to question 63)	1) Once a year	2) Twice a year
3) Three times a year	4) Four or more times a year	

62. Type of herbicide containers, and disposal method (Mark with an X)

Container type:

1) Glass	2) Aluminum	3) Sheet metal
4) PET	5) Plastic	6) Other

Disposal method:

1) Trash	2) Open dump	3) Special deposit sites	4) Left in the field
5) Reused	6) Burned	7) Other	

63. How often do you use pesticides per year? (Mark with an X)

0) Does not use (skip to question 65)	1) Once a year	2) Twice a year
3) Three times a year	4) Four or more times a year	

64. Type of pesticide containers, and disposal method (Mark with an X)

Container type:

1) Glass	2) Aluminum	3) Sheet metal
4) PET	5) Plastic	6) Other

Disposal method:

1) Trash	2) Open dump	3) Special deposit sites	4) Left in the field
5) Reused	6) Burned	7) Other	

3. Use of machinery and energy for forage maintenance and production

65. Type of irrigation (Mark with an X)

1) Rainfed (seasonal)	2) Canal	3) Drip irrigation
4) Sprinkler	5) Other	

66. Type of machinery and equipment (Mark with an X)

0) No machinery or equipment	1) Tractor	2) Trailers	3) Plow
4) Machete	5) Mower/chopper	6) Pump	7) Other

4. Use of machinery and energy for milk production

67. Where do you milk the cows? (Mark with an X)

1) Milking parlor	2) Handling corral	3) Field	4) Other

68. Machinery and equipment used for milk (Mark with an X)

0) None	1) Cooling tank	2) Curd vat
3) Cream separator	4) Other	

69. Type of milking (Mark with an X)
   1) Mechanical: Electric ( ) Gasoline-powered ( ) Mixed ( ) 2) Manual

70. What container do you use for the milk? (Mark with an X)

1) Plastic	2) Metal	3) Aluminum	4) Other

71. How do you transport the milk to the delivery point? (Mark with an X)

1) On foot	2) By horse	3) Motorcycle
4) Pickup truck	5) Other	

5. Environmental impacts

72. Environmental constraints (Mark with an X)

0) None	1) Droughts	2) Floods
3) Low soil fertility	4) Low water availability	5) "Nortes" (cold fronts)
6) Pests	7) High temperatures	8) Other

73. Which months do you consider the dry season? (Mark with an X)

Jan	Feb	Mar	Apr	May	Jun	Jul	Aug	Sep	Oct	Nov	Dec

How does it affect your animals? (Mark with an X)

0) None	1) Reduced production	2) Weight loss
3) They get sick	4) They fail to conceive / do not come into heat	5) Death of animals
6) Other		

74. Which months do you consider the rainy season? (Mark with an X)

Jan	Feb	Mar	Apr	May	Jun	Jul	Aug	Sep	Oct	Nov	Dec

How does it affect your animals? (Mark with an X)

0) None	1) Reduced production	2) Weight loss
3) They get sick	4) They fail to conceive / do not come into heat	5) Death of animals
6) Other		

75. Which months do you consider "nortes" (cold-front) season? (Mark with an X)

Jan	Feb	Mar	Apr	May	Jun	Jul	Aug	Sep	Oct	Nov	Dec

How does it affect your animals? (Mark with an X)

0) None	1) Reduced production	2) Weight loss
3) They get sick	4) They fail to conceive / do not come into heat	5) Death of animals

6) Other		

6. Animal health and welfare

76. Do you consider that your cows eat well? Yes ( ) No ( )

77. Do you consider that your cows are well cared for? Yes ( ) No ( )

78. Do you consider that your cows live well? Yes ( ) No ( )

79. Do you have enough water for the animals? Yes ( ) No ( )

80. Where do you source water for the cows? (Mark with an X)

1) Piped/drinking water	2) Well	3) Pond (jagüey)
4) River/stream	5) Other	

81. How do you bring water to the animals? (Mark with an X)

1) Pump	2) Vehicle	3) Bucket	4) Other

82. Do you deworm the animals? Internal: Yes ( ) No ( ) External: Yes ( ) No ( )

83. What are the most common diseases in your herd, and how are they treated? (Mark with an X)

Disease	How is it treated?
1) Mastitis	
2) Pneumonia	
3) Stomatitis	
4) Piroplasmosis	
5) Anaplasmosis	
6) Rabies ("derriengue")	
7) Bloat	
8) Foot rot	
9) Tuberculosis	
10) Brucellosis	
11) Other	

Treatment options: 0) None 1) Antibiotic 2) Home remedies 3) Veterinary care 4) Other

84. What do you do with milk from cows under treatment? (Mark with an X)

0) Does not milk cows under treatment	1) It is sold	2) Calves consume it
3) Poured into the drainage/sewer	4) Poured in the field/yard	5) Consumed by other species
6) Poured into streams or water bodies	7) Other	

85. Do you divide your animals into production-stage groups (e.g., productive animals, calves)? Yes ( ) No ( )

86. How do you control ticks? (Mark with an X)

0) No ticks	1) Not controlled	2) Chemical products
3) Stocking-rate control	4) Controlling pasture growth	5) Resistance testing
6) Other		

Supplementary Material S1: Questionnaire used for data collection

IV. SOCIAL

1. Decent living / quality of life

87. You have access to: (Mark with an X)

0) None	1) Sufficient food	2) Health services
3) Housing	4) Clothing	5) Education
6) Transportation	7) Media/communication (TV, cell phone, internet, etc.)	8) Entertainment
9) Retirement or pension plan	10) Security	11) Personal loans/credit
12) Public services (electricity, water, drainage)	13) Other	

88. What is the money obtained from production spent on? (Mark with an X)

1) Another economic activity	2) Reinvestment	3) Food	4) Education
5) Entertainment	6) Transportation	7) Clothing	8) Payment of services

2. Organization

89. Are you part of any organization? If not, why? Have you held any position (please specify which)? ________________

0) No	1) Local livestock association	2) Regional livestock association
3) State livestock association	4) Producers' association	5) Informal groups
6) Other		

90. What benefits have you obtained from this organization? (Mark with an X)

0) None	1) Access to courses	2) Selling your products	3) Access to credit
4) Advisory services	5) Government support	6) Information exchange	7) Other

91. What is the means of information exchange among producers? (Mark with an X)

0) None	1) In-person meetings	2) WhatsApp
3) Facebook	4) Video conference	5) Other

92. How do you relate to your buyer? (Mark with an X)

0) None	1) By contract	2) Captive/exclusive relationship	3) Sells to best bidder
4) Other			

93. What is the destination of the milk production? (Mark with an X)

1) Final client / consumer	2) Collector/deliverer	3) Wholesaler (e.g., Liconsa)

94. Who do you sell the milk to? ________________________

95. What other marketing options would you like to have? (You may select more than one)

0) Not interested in other options	1) Sell to Liconsa	2) Sell to Nestlé
3) Supermarket sale	4) Direct sale to final consumer	5) Other

96. Have you considered options to increase your income? (Mark with an X)

0) None	1) Make cheese	2) Change productive species

3) Change economic activity	4) Improve genetics	5) Other

97. How is your ranch organized? (Mark with an X)

1) The owner	2) Paid family members	3) Unpaid family members	4) Permanent workers
5) Administrative staff	6) Hired workers	7) Temporary workers	8) Other

3. Employment generation

98. How many employees do you have? ____________ No ( ) (if none, skip to question 108)

99. Type of payment is by (Mark one or more with an X)

1) Day	2) Week	3) Month	4) Other

100. How much do you pay per day? ____________

101. How many hours a day do the employees work? ____________

102. Your workers are (Mark with an X)

1) Neighbors from the locality	2) From the municipality	3) From another municipality
4) From another State	5) From another country	6) Other

103. What activities do these employees carry out? (Mark with an X)

1) Milking	2) General cattle handling	3) Field work
4) All of the above	5) Other	

104. Do you have problems finding labor in the area? Yes ( ) No ( )

105. Do you consider that the people who work with you are satisfied? Yes ( ) No ( )

106. Do your workers carry out their duties without needing you to be present on the ranch? Yes ( ) No ( )

107. Your workers have access to: (Mark with an X)

0) I don't know	1) Good pay	2) Good treatment
3) Transportation support	4) Lodging provided	5) Food provided
6) Other		

V. GOVERNANCE OF THE PRODUCTION SYSTEM

1. Participation and succession planning

108. Which vaccination/health campaigns have you participated in? (Mark with an X)

0) None	1) Brucellosis	2) Tuberculosis
3) Both	4) Other	

109. How many people make up your household? ____Women ____Men ____

110. How many people are economically dependent on you? ____________

111. How many household members work on the ranch or plot? ____________

112. Was your father, or any other family member, a livestock producer? Yes ( ) No ( )

113. Do you have children? If so, please complete the following table:

Supplementary Material S1: Questionnaire used for data collection

Sex (1. Female 2. Male)	Age (years)	Education (0. None 1. Primary 2. Secondary 3. High school 4. University)	Helps with ranch work (1. Yes 2. No)	Activity performed

Activity performed: 1) Milking 2) General cattle handling 3) Field work 4) Ranch administration 5) All of the above 6) Other

114. Are your children interested in continuing with livestock farming? Yes ( ) No ( )

115. Why? ____

2. Decision-making

116. Who makes the production decisions? (Mark with an X)

1) You alone	2) You and your spouse	3) You and your family	4) You and your workers

117. If you are not present, who makes the decisions (when necessary)? _______________________

118. Do you take your workers' opinions into account in decision-making? (Mark with an X)

1) I don't consider it	2) On general cattle handling	3) On field work	4) Other

3. Equity

119. Do you have a preference for hiring men or women on your ranch? (Mark with an X)

Activity	1) Man	2) Woman	3) Either
1) Milking			
2) General handling			
3) Administrative			
4) Marketing			

120. What decisions do you share with your wife? (Mark with an X)

0) I have no wife (skip to question 122)	1) None	2) Buying and selling livestock
3) Ranch administration	4) Hiring staff	5) All of the above

121. Does your wife receive a salary for her work? Yes ( ) No ( )

4. Accountability

122. Are you interested in producing with quality? Yes ( ) No ( )

123. What is the reason? (Mark with an X)

1) Improve price	2) Have a better product	3) Other

124. Do you know the legislation governing milk production? Yes ( ) No ( )

125. Has milk been returned to you for not meeting the buyer's quality requirements? (Mark with an X)

1) Never	2) Sour/spoiled milk	3) Watered down
4) Low fat content	5) Other	

126. What do your clients think of the milk? (Mark with an X)

1) Good	2) Bad	3) Fair

127. Would they consider paying more for milk quality and/or composition? Yes ( ) No ( )

128. Milk payment is made (Mark with an X)

1) Daily	2) Weekly	3) Monthly	4) Other

129. What do your neighbors think of your ranch? (Mark with an X)

0) Nothing in particular	1) Good management	2) Not comfortable with my activity
3) Other		

130. Have you received complaints from neighbors or others about the management of your animals or plot? Yes ( ) No ( )

5. Efficiency

131. Are you part of SADER's Single Registry of Beneficiaries ("Padrón Único de Beneficiarios")? Yes ( ) No ( )

132. Have you applied for any credit for your production? Yes ( ) No ( )

Type of credit	Institution
1) Working capital (raw materials, supplies, and operating expenses)	
2) Capital/equipment loan (to acquire, replace, or purchase machinery, equipment, or livestock)	

133. Have you received technical assistance? Yes ( ) From whom? ____________ No ( )

134. Have you received any courses? (Mark with an X)

0) None	1) Milk quality	2) Forage production
3) Accounting	4) Reproduction	5) Administration
6) Feeding	7) Tick control	8) Other

135. What courses would you like to receive? (Mark with an X)

0) None	1) Milk quality	2) Forage production
3) Accounting	4) Reproduction	5) Administration
6) Feeding	7) Tick control	8) Other

136. Are you interested in a production model that would allow you to increase your profits? Yes ( ) No ( )

137. Have you received support from: (Mark with an X)

0) No one	1) Municipal government	2) State government
3) Federal government	4) Private companies	5) Non-Governmental Organization (NGO)
6) Other		

Final comments: ____

____

____

____

Producer's name and signature

(confirms that they were visited and interviewed) (Optional)

____

Interviewer's name and signature

Supplementary Material S2: Definition, coding, and aggregation rules of questionnaire-derived indicators.

Composite indicator development

To characterize farm management, forage resources, biological diversity, and producer-reported environmental and animal health conditions, a set of composite indicators was developed from information collected through structured farm surveys. Each indicator was constructed from binary variables, assigning a value of 1 when the characteristic or condition was present and 0 when it was absent.

For each indicator, binary scores were summed to obtain a raw score. All components were assigned equal weight because no empirical evidence or theoretical justification supported differential weighting among variables. Since the indicators comprised different numbers of components, raw scores were standardized to a common scale ranging from 1 (lowest expression of the measured attribute) to 10 (highest expression of the measured attribute) using the following equation:

$$I_i = 1 + 9\left(\frac{R_i}{R_{\max}}\right)$$

where I_i is the standardized indicator for farm i, R_i is the raw score, and $R_{\max}$ is the maximum possible raw score for the corresponding indicator.

The indicators were constructed using a fully compensatory additive aggregation approach, assuming that all components contributed equally to the measured attribute.

Soil Management Indicator (SMI)

The Soil Management Indicator (SMI) quantifies the adoption of soil conservation and management practices at the farm level. The indicator comprises ten practices: (i) manure incorporation, (ii) implementation of silvopastoral systems, (iii) pasture rotation, (iv) temporary grazing exclusion using electric fencing, (v) pasture rehabilitation, (vi) maintenance of vegetation cover, (vii) adjustment of stocking rates according to paddock carrying capacity, (viii) establishment of live fences, (ix) chemical fertilization, and (x) other soil conservation practices. The raw score ranges from 0 to 10, with higher values indicating greater implementation of soil conservation and management practices.

Pasture Availability Indicator (PAI)

The Pasture Availability Indicator (PAI) measures the temporal availability of forage throughout the year. Pasture availability was recorded for each month of the year, resulting in a raw score ranging from 0 to 12. Higher scores indicate greater year-round pasture availability.

Pasture Species Diversity (PSD)

The Pasture Species Diversity (PSD) quantifies the richness of cultivated pasture species present on the farm. The indicator considers the presence of *Cynodon plectostachyus*, *Digitaria eriantha*, *Brachiaria dictyoneura*, *Brachiaria brizantha*, *Hyparrhenia rufa*, *Brachiaria humidicola*, *Megathyrsus maximus*, *Pennisetum purpureum*, *Brachiaria decumbens*, *Brachiaria* hybrid, *Brachiaria* Cobra hybrid, and other cultivated pasture

species. The raw score ranges from 0 to 12, with higher values indicating greater pasture species richness.

Tree Species Diversity (TSD)

The Tree Species Diversity (TSD) quantifies the diversity of tree functional groups present on the farm. Five groups were considered: forage trees, fruit trees, shade trees, timber trees, and other tree species. The raw score ranges from 0 to 5, with higher values indicating greater diversity of tree functional groups.

Domestic Animal Diversity Indicator (PLS)

The Domestic Animal Diversity Indicator (PLS) quantifies the diversity of productive and working domestic animal species present on the farm. The indicator includes poultry, pigs, sheep, horses, donkeys, mules, bees, and other domestic animal species. The raw score ranges from 0 to 8, with higher values indicating greater domestic animal diversity.

Wildlife Richness Indicator (OWDI)

The Wildlife Indicator (OWDI) quantifies the occurrence of wildlife groups observed on the farm. The indicator includes birds, reptiles (iguanas, lizards and snakes), rodents, bats, beetles and other wildlife groups. The raw score ranges from 0 to 8, with higher values indicating greater wildlife occurrence.

Rainfall and Flooding Impact Indicator (DIR)

The Rainfall and Flooding Impact Indicator (DIR) quantifies the occurrence of producer-reported adverse impacts associated with excessive rainfall or flooding throughout the year. Impacts were recorded monthly, resulting in a raw score ranging from 0 to 12. Higher scores indicate greater annual exposure to rainfall- and flooding-related impacts.

North Wind Impact Indicator (DINWI)

The North Wind Impact Indicator (DINWI) measures the annual occurrence of adverse impacts attributed to north winds as reported by producers. For each month, the occurrence of north wind-related impacts was recorded as a binary variable (1 = impact reported; 0 = no impact reported). Monthly values were summed to obtain a raw score ranging from 0 to 12 and subsequently standardized to a common scale from 1 to 10. Higher values indicate greater producer-reported exposure to adverse impacts associated with north winds.

Animal Health Indicator (DILD)

The Animal Health Indicator (DILD) quantifies the occurrence of producer-reported diseases and health-related incidents affecting livestock. The indicator was calculated by summing the reported disease and health incident categories. Higher scores indicate poorer animal health conditions.

Supplementary Material S3: Additional statistical analyses supporting the robustness and characterization of the farm typologies.

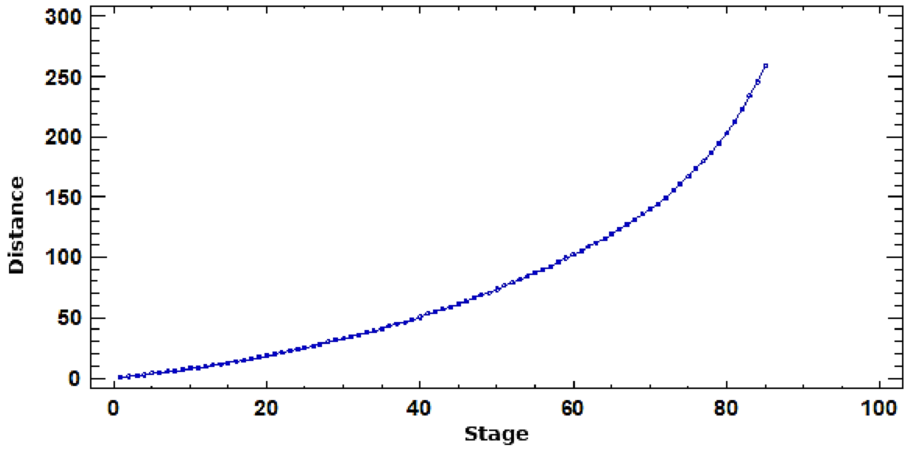

Figure S1. Agglomeration-distance plot obtained from hierarchical cluster analysis using Euclidean/squared Euclidean distance.
